# Symptom prevalence differences of depression as measured by BDI and PHQ scales in the Look AHEAD study

**DOI:** 10.1002/osp4.378

**Published:** 2019-12-19

**Authors:** Elizabeth M. Vaughan, Craig A. Johnston, Jennette P. Moreno, Lawrence J. Cheskin, Gareth R. Dutton, Molly Gee, Sarah A. Gaussoin, William C. Knowler, W. Jack Rejeski, Thomas A. Wadden, Susan Z. Yanovski, John P. Foreyt, The Look AHEAD Research Group

**Affiliations:** ^1^ Department of Medicine Baylor College of Medicine Houston Texas; ^2^ Department of Health and Human Performance University of Houston Houston Texas; ^3^ USDA/ARS Children's Nutrition Research Center, Department of Pediatrics‐Nutrition Baylor College of Medicine Houston Texas; ^4^ Department of Nutrition and Food Studies, College of Health and Human Services George Mason University Fairfax Virginia; ^5^ Department of Health, Behavior & Society Johns Hopkins Bloomberg School of Public Health Baltimore Maryland; ^6^ Division of Preventive Medicine University of Alabama at Birmingham Birmingham Alabama; ^7^ Department of Biostatistical Sciences and Data Science, Division of Public Health Services Wake Forest School of Medicine Winston‐Salem North Carolina; ^8^ Diabetes Epidemiology and Clinical Research Section National Institute of Diabetes and Digestive and Kidney Diseases, National Institutes of Health Phoenix Arizona; ^9^ Division of Public Health Sciences Wake Forest School of Medicine Winston‐Salem North Carolina; ^10^ Department of Psychiatry Perelman School of Medicine, University of Pennsylvania Philadelphia Pennsylvania; ^11^ Division of Digestive Diseases and Nutrition National Institute of Diabetes and Digestive and Kidney Diseases Bethesda Maryland

**Keywords:** depression, diabetes, obesity, screening

## Abstract

**Objective:**

To compare depressive symptomatology as assessed by two frequently used measures, the Beck Depression Inventory (BDI‐1A) and Patient Health Questionnaire (PHQ‐9).

**Methods:**

Investigators conducted a cross‐sectional secondary analysis of data collected as part of the follow‐up observational phase of the Look AHEAD study. Rates of agreement between the BDI‐1A and PHQ‐9 were calculated, and multivariable logistic regression was used to examine the relationship between differing depression category classifications and demographic factors (ie, age, sex, race/ethnicity) or comorbidities (ie, diabetes control, cardiovascular disease).

**Results:**

A high level of agreement (κ = 0.47, 95% CI (0.43 to 0.50)) was found in the level of depressive symptomatology between the BDI‐1A and PHQ‐9. Differing classifications (minimal, mild, moderate, and severe) occurred in 16.8% of the sample. Higher scores on the somatic subscale of the BDI‐1A were significantly associated with disagreement as were having a history of cardiovascular disease, lower health‐related quality of life, and minority racial/ethnic classification.

**Conclusions:**

Either the BDI‐1A or PHQ‐9 can be used to assess depressive symptomatology in adults with overweight/obesity and type 2 diabetes. However, further assessment should be considered in those with related somatic symptoms, decreased quality of life, and in racial/ethnic minority populations.

## 1 INTRODUCTION

Depression is 60% more common in individuals with type 2 diabetes than in the general population and is associated with numerous poor health behaviors including physical inactivity and higher dietary fat intake.^1^, ^2^ Individuals suffering from depression are at increased risk of adverse cardiovascular outcomes in addition to workplace absenteeism, unemployment, and disability.^3^, ^4^, ^5^ Depression increases the activation of the hypothalamic‐pituitary‐adrenal axis, sympathetic nervous system, and proinflammatory cytokines, resulting in elevated serum glucose levels.^6^ However, only half of patients with diabetes and major depression are recognized as depressed by their primary care provider.^7^ This may relate to these conditions’ overlapping symptoms including fatigue, appetite changes, and decreased libido.^8^, ^9^ Brief and easy‐to‐administer screening questionnaires for individuals with diabetes are critical to improve surveillance of depression.^10^

Historically, the 21‐item Beck Depression Inventory (BDI) has been the most widely used instrument in healthcare for assessing symptoms of depression.^11^ The BDI (BDI, BDI‐1A, BDI‐11) presents individuals with a series of statements consistent with depressive symptoms and asks to choose the one closest to how they felt, using both cognitive and somatic variables (eg, mood, muscle aches).^11^ However, several barriers in the BDI's use have been noted, including high item difficulty (skill/ability needed to complete an item).^12^ In addition, shorter versions (eg, 16‐item), though less time consuming, have a lower sensitivity in identifying true cases of depression.^13^

A shorter instrument, the Patient Health Questionnaire (PHQ‐9), was developed to reduce the time required for completion and is now widely used in clinical settings.^14^, ^15^ Both the BDI and PHQ‐9 are self‐report instruments that use 4‐point Likert‐type scales to assess symptoms of depression and are well‐validated measures.^11^, ^14^, ^15^, ^16^ A unique characteristic of the PHQ is its inclusion of the criteria for depression stipulated by the Diagnostic and Statistical Manual (ie, DSM‐IV, DSM‐5).^17^ The PHQ‐9 asks individuals to read each of the nine criteria of a major depressive episode from the DSM and indicate how often they have felt that way in the last two weeks.^14^, ^15^ Some investigators argue that this direct mapping to the DSM, along with the PHQ‐9's shorter length, makes it a potentially more desirable measure for screening for depression.^14^, ^17^, ^18^ Although the BDI and PHQ‐9 have been used for measuring symptoms of depression in individuals with type 2 diabetes and have been found to be effective,^7^, ^19^, ^20^, ^21^ there are no studies that compare these instruments in a population with type 2 diabetes. The PHQ‐9 has increased in use and efficacy data are needed to ensure it is the appropriate tool to use in with this patient population.

The Look AHEAD (Action for Health in Diabetes) trial, a randomized controlled trial comparing intensive lifestyle intervention (ILI) to diabetes support and education (DSE) in adults with overweight/obesity and type 2 diabetes, offered a unique opportunity to compare the BDI and the PHQ‐9 in this population.^22^, ^23^ While the active intervention phase of Look AHEAD has concluded, an observational cohort continues to be followed. The objectives of this study were to compare depressive symptomatology as assessed by the BDI‐1A and PHQ‐9 for Look AHEAD participants in the observational follow‐up cohort. Specifically, investigators compared the BDI‐1A and PHQ‐9 in the following areas: (1) depression prevalence using both instruments’ cut points for no (BDI‐1A)/minimal (PHQ‐9), mild, moderate, and severe depression, (2) differences in degree of reported depressive symptomatology, (3) differences in degree of reported depressive symptomatology based on demographic factors (ie, age, sex, race/ethnicity) or comorbidities (diabetes control, cardiovascular disease [CVD]). In addition, investigators examined if the BDI‐1A's cognitive and somatic scales explain any observed differences. Investigators hypothesized that the BDI‐1A and PHQ‐9 can be used interchangeably for depression screening for adults with overweight/obesity and type 2 diabetes.

## 2 METHODS

Look AHEAD was a randomized controlled trial that examined the impact of participation in a long‐term ILI designed to produce weight loss on the health outcomes of 5145 adults with overweight/obesity and type 2 diabetes. Participants were randomized to one of two conditions: ILI or DSE (control group).^23^ Its principal goal was to determine the impact of the ILI on cardiovascular morbidity and mortality (ie, nonfatal and fatal myocardial infarction and stroke, hospitalization for angina). The design and methods of the trial have been described in detail elsewhere.^23^, ^24^ Despite greater weight loss in the ILI than the DSE group at every annual assessment, the Look AHEAD intervention was stopped at a median follow‐up of 9.6 years due to a lack of significant differences in cardiovascular‐related morbidity or mortality between the two conditions.^25^

All living participants at the end of the trial were invited to join a follow‐up observational study to determine the longer‐term effects of the intervention on several outcomes. Of the 3985 individuals who attended a follow‐up clinic examination, 3703 had both the BDI‐1. A and PHQ‐9 performed and their results are the subject of these analyses (Table 1). During the original trial, depressive symptomatology was measured annually with the BDI‐1A, whereas in the follow‐up observational study, its symptomatology was measured once with both the BDI‐1A and PHQ‐9. The current analysis considers only responses to the BDI‐1A and PHQ‐9 questionnaires. Data were collected from 2013 to 2014.

**Table 1:** Baseline characteristics of Look AHEAD participants in the follow‐up observational study with both BDI‐1A and PHQ‐9 data (*n* = 3703)

Variable	
**Age**	**Mean (SD)**
	69.79 (6.37)
	**N (%)**
**Original treatment assignment**	
DSE	1841 (49.72)
ILI	1862 (50.28)
**Sex**	
Women	2253 (60.84)
**Race/Ethnicity**	
African American or Black (not Hispanic)	618 (16.69)
American Indian, Native American, Alaskan Native	160 (4.32)
Asian or Pacific Islander	41 (1.11)
Caucasian	2305 (62.25)
Hispanic	497 (13.42)
Other or Mixed	82 (2.21)
**Education**	
<13 years	1360 (37.63)
13‐16 years	725 (20.06)
>16 years	1529 (42.31)
**Weight (kg)**	94.30 (19.47)
**BMI (kg/m** ^**2**^ **)**	34.51 (7.63)
**History of CVD**	
Yes	967 (26.11)
No	2736 (73.89)
**Diabetes medications**	
None	474 (14.82)
Oral only	1420 (44.39)
Insulin only	415 (12.97)
Insulin and oral	890 (27.82)
**HbA1c (%)**	7.45 (1.55)
**Antidepressant use**	
Yes	577 (16.16)
No	2994 (83.84)
**BDI score** *	5.27 (5.26)
**BDI score – cognitive subscale**	1.92 (3.06)
**BDI score – somatic subscale**	3.35 (2.82)
**PHQ score** *	2.75 (4.38)
**History of depression**	
Yes	750 (20.26%)
No	2951 (79.74%)
**SF‐36 mental component summary**	54.20 (9.55)
**SF‐36 physical component summary**	42.88 (10.30)
**SF‐36 general health**	45.83 (9.60)
**Binge eating disorder**	
Yes	488 (13.24)
No	3199 (86.76)

* Range of scores: BDI 0‐44, PHQ9 0‐26, SF‐36 MCS 4.16‐76.23, SF‐36 PCS 8.96‐66.12, SF‐36 general health 16.23‐63.90.

*Abbreviations: BDI, Beck Depression Inventory; CVD, cardiovascular disease; DSE, diabetes support and education; ILI, intensive lifestyle intervention; MCS, mental component summary; PHQ, Patient Health Questionnaire; SF, Medical Outcomes Short Form; SD, standard deviation.

### 2.1 Outcome measures

#### 2.1.1 Patient Health Questionnaire

The version of the PHQ used in Look AHEAD was the PHQ‐9, a 9‐item self‐report measure of depressive symptoms in adults that corresponds directly with DSM criteria for a major depressive episode.^14^, ^15^ Participants indicated, using 0 to 3 scales, how frequently they experienced each of nine symptoms of depression in the past two weeks, resulting in composite scores ranging from 0 to 27. The PHQ‐9 classifies individuals as having no symptoms of depression (ie, score of 0 to 4), mild symptoms (ie, score of 5 to 9), moderate symptoms (ie, score of 10 to 14), moderately severe (ie, score 15 to 19), or severe symptoms (ie, score of 20 to 27).^14^, ^15^ In the current analyses, the moderate and moderately severe categories were combined to more closely map onto the four categories of the BDI‐1A.

#### 2.1.2 Beck Depression Inventory

The version of the BDI used in Look AHEAD was the BDI‐IA, a well‐validated, 21‐item, self‐report measure of depressive symptoms in adults.^11^ Participants were asked to respond to items based on how they had been feeling in the past week. Each item has four answer choices of increasing severity scored on a scale of 0 to 3, resulting in total scores ranging from 0 to 63. Higher scores indicate greater severity of symptoms. Participants were identified as having minimal symptoms of depression (ie, score of 0 to 9), mild symptoms (ie, score of 10 to 17), moderate symptoms (ie, score of 18 to 29), or severe symptoms (ie, score of 30 to 63).^11^ These same classification categories were used in these analyses. The BDI‐1A contains two subscales that primarily measure somatic (7 items) and cognitive symptoms (14 items) of depression. The BDI‐1A and PHQ‐9 use the same names for all depression categories (ie, mild, moderate, severe) except for the lowest category of depression where the BDI‐1A label is *minimal* depression and the PHQ‐9 label is *no* depression. Although the BDI‐1A and PHQ‐9 were measured more than once during Look AHEAD, they were collected simultaneously at only one time point (beginning of observational phase), and thus longitudinal evaluations were not feasible.

### 2.2 Other measures

Participants provided basic demographic information including sex, race/ethnicity, and history of CVD or depression through questionnaires. The Medical Outcomes Short Form 36 (SF‐36) was used to assess health‐related quality of life,^26^ and both a physical component summary (PCS) score and mental component summary (MCS) score were calculated from this measure. Binge eating disorder was assessed through a self‐report measure based on the Questionnaire on Eating and Weight Patterns.^27^ Hemoglobin A1c (HbA1c) levels were drawn at the study site. Hypoglycemic agent and antidepressant use were collected by medications brought to study site by participant. All measures, including the BDI‐1A and PHQ‐9, were collected at the same visit.

### 2.3 Statistical analyses

Investigators assessed the relationship between certain variables of interest and differing depression category classifications using multivariable logistic regression. The differences of BDI‐1A and PHQ‐9 scales prevented a nonbinary logistic regression analysis agreement classification. Variables included age, treatment assignment, sex, race/ethnicity, education, weight, body mass index (BMI), CVD history, diabetes medications, HbA1c, antidepressant (AD) use, history of depression, SF‐36, binge eating disorder, and the BDI‐1A total score as well as the cognitive and somatic subscales. The variables chosen to be associated with nonagreement between the BDI and the PHQ were based on published literature in adults with overweight/obesity diagnosed with depression.^22^, ^28^ Treatment assignment was included in the current study model since previous investigators found a significant treatment effect related to depression and quality of life.^22^ Authors controlled for treatment assignment to remove difference that is related to treatment differences as opposed to nonagreement. Analyses included all study participants who had data for both BDI‐1A and PHQ‐9 and were performed using SAS version 9.4 (SAS Institute, Cary, NC, USA).

## 3 RESULTS

### 3.1 Categorization of depressive symptomatology by the BDI‐1A and PHQ‐9

A descriptive inspection of differences in reporting of symptomatology indicated that 85.9% of participants were categorized as having no or minimal depressive symptoms by the BDI‐1A compared with 82.1% by the PHQ‐9. The BDI‐1A classified 11.8% of participants as having mild/moderate symptoms compared to 14.1% by the PHQ‐9. A total of 2.0% of participants were classified as having moderate/moderately severe symptomatology by the BDI‐1A compared to 3.6% by the PHQ‐9. The percentage of individuals classified in the severe range was 0.3% and 0.2% by the BDI‐1A and PHQ‐9, respectively.

### 3.2 Disagreement in the categorization of depressive symptomatology

From the 3985 participants, 3703 individuals had data from both the BDI‐1A and PHQ‐9 (Table 1). A total of 622 (16.8%) participants had conflicting levels of depression classification between the PHQ‐9 and BDI‐1A. Table 2 shows where these differences occur. For 419 (11.3%) participants, the PHQ‐9 classified at a higher depression level than the BDI‐1A, and in 203 (5.5%), the reverse was true. The kappa statistic for agreement was 0.47 (95% CI = 0.43 to 0.50). Agreement was greater if the scores were dichotomized as none/minimal vs mild through severe (BDI‐1A) and no/minimal vs mild through severe (PHQ‐9), in which case κ = 0.56 (95% CI = 0.53 to 0.60).

**Table 2:** Differing depression classifications between BDI‐1A and PHQ‐9 (*n* = 3703)

	PHQ classifications			
BDI classifications	No/minimal* (%)	Mild (%)	Moderate/Moderately severe (%)	Severe (%)
**None/minimal**	2772 (74.8)	302 (8.16)	23 (0.62)	1 (0.03)
**Mild**	156 (4.21)	246 (6.64)	84 (2.27)	1 (0.03)
**Moderate/Moderately severe**	8 (0.22)	28 (0.76)	58 (1.57)	8 (0.22)
**Severe**	0 (0.00)	1 (0.03)	10 (0.27)	5 (0.14)

* The lowest score on the BDI (0‐9) is classified as *minimal* depression, whereas the lowest score on the PHQ^1^, ^2^, ^3^, ^4^ is classified as *no* depression. All other categories are classified with the same labels (ie, mild, moderate, severe).

*Abbreviations: BDI, Beck Depression Inventory; PHQ, Patient Health Questionnaire.

### 3.3 Factors associated with disagreement in categorization

An initial multivariable logistic regression analysis revealed that higher scores on the BDI‐1A were associated with disagreement in depression category classification (OR = 1.106 (95% CI 1.077, 1.136) p <0.0001). To gain understanding of differences found based on BDI‐1A score, a refined multivariable logistic regression analysis was conducted using the cognitive and somatic subscales of the BDI‐1A modeling the probability of differing classifications between the BDI‐1A and PHQ‐9. This model indicated that higher scores on the somatic scale of the BDI‐1A were associated with disagreement in depression category classification (OR = 1.190 (95% CI 1.131, 1.253), p <0.0001), and higher scores on the cognitive scale were not significantly associated with disagreement in depression category classification (OR = 1.042 (95% CI 0.997, 1.089), p = 0.0659). Other factors that were related to an increased likelihood of differing classification between the BDI‐1A and PHQ‐9 included having a history of CVD, a lower MCS score as measured by the SF‐36, a lower PCS score as measured by the SF‐36, and being Hispanic or American Indian/Alaskan Native as compared to Caucasian . Results from the refined multivariable logistic regression analysis are listed in Table 3.

**Table 3:** Multivariate logistic regression analyses to determine factors associated with disagreement between the BDI‐1A and PHQ‐9 (n=622)

Variable	Odds ratio (95% Confidence interval)	*p*‐value
**Age**	1.008 (0.99, 1.03)	0.42
**Treatment assignment**		0.80
DSE	0.972 (0.77, 1.22)	
ILI	1.00	
**Sex**		0.07
Women	1.00	
**Race/ethnicity**		0.01
African American or non‐Hispanic Black	1.098 (0.79, 1.53)	
American Indian, Native American, Alaskan Native	2.025 (1.17, 3.50)	
Asian or Pacific Islander	1.070 (0.35, 3.28)	
Hispanic	1.547 (1.10, 2.17)	
Other or Mixed	0.403 (0.15, 1.07)	
Caucasian	1.00	
**Education**		0.93
<13 years	0.977 (0.71, 1.35)	
13‐16 years	0.951 (0.73, 1.24)	
>16 years	1.00	
**Weight (kg)**	1.001 (0.99, 1.01)	0.83
**BMI (kg/m** ^**2**^ **)**	1.006 (0.99, 1.03)	0.59
**History of CVD**		0.003
Yes	1.466 (1.14, 1.90)	
No	1.00	
**Diabetes medications**		0.20
None	0.694 (0.50, 0.99)	
Oral only	0.773 (0.51, 1.18)	
Insulin only	0.753 (0.52, 1.09)	
Insulin and oral	1.00	
**HbA1c (%)**	1.013 (0.94, 1.10)	0.75
**Antidepressant use**		0.11
Yes	1.319 (0.94, 1.85)	
No	1.00	
**BDI score – cognitive subscale**	1.042 (1.00, 1.09)	0.066
**BDI score – somatic subscale**	1.190 (1.13, 1.25)	<0.0001
**History of depression**		0.73
Yes	0.946 (0.69, 1.30)	
No	1.00	
**SF‐36 mental component summary**	0.949 (0.936, 0.962)	<0.0001
**SF‐36 physical component summary**	0.967 (0.95, 0.98)	<0.0001
**General health score**	0.989 (0.97, 1.01)	0.2169
**Binge eating disorder**		0.25
Yes	1.199 (0.88, 1.64)	
No	1.00	

*Abbreviations: BDI, Beck Depression Inventory; CVD, cardiovascular disease; DSE, diabetes support and education; ILI, intensive lifestyle intervention; MCS, mental component summary; PHQ, Patient Health Questionnaire; SF, Medical Outcomes Short Form.

## 4 DISCUSSION

Investigators conducted a novel comparison of the BDI‐1A and PHQ‐9 as screening measures for depression in adults with overweight/obesity and type 2 diabetes. Comparisons revealed a high level of agreement (83%) between measures (κ=0.47) in the classification of depressive symptoms. These results are similar to those found in studies that have compared the BDI‐1A and PHQ‐9 in other disease states with kappa's ranging from 0.24 to 0.64.^29^, ^30^, ^31^, ^32^ Although the BDI‐1A has historically been considered the optimal measure of depressive symptoms, the PHQ‐9 is briefer and available at no cost, which may be advantageous to many healthcare providers and particularly those working with resource‐limited populations. This moderate level of agreement suggests that either the BDI‐1A or PHQ‐9 may be used when assessing depressive symptoms in most adults with overweight/obesity and type 2 diabetes. If the questionnaire results are dichotomized into those indicating no depression vs higher levels (possibly prompting further clinical evaluation), the agreement is better (κ=0.56), although still not ideal.

Although the rate of agreement between these two measures suggests a degree of interchangeability, it should be noted that the BDI‐1A and PHQ‐9 yielded conflicting levels of depression for a substantial proportion of individuals (17%). In general, participants were more likely to be classified as depressed or having a greater severity of depressive symptoms by the PHQ‐9 as compared to the BDI‐1A. For example, 8% of participants were classified as having mild depression by the PHQ‐9 when the BDI‐1A reflected minimal or no symptoms of depression. Conversely, only 4% of individuals were identified as having mild depression by the BDI‐1A and minimal or no depressive symptoms by the PHQ‐9. As suggested by other studies, this may be due to the lower cutoff scores used by the PHQ‐9.^29^ As a result, the PHQ‐9 is more likely to pick up minimal levels of depression, which may lead to earlier identification of depressive symptoms. This may make the PHQ‐9 a more desirable measure in individuals who are at higher risk of complications associated with depression, such as medication nonadherence.^33^ However, less discrepancy between instruments is found in the moderate to severe ranges.

In these analyses, the most common type of disagreement between measures was identification as mildly depressed on one measure and minimal or no symptoms on the other. Few (1% of cases) participants had a moderate or severe level of depression indicated by one measure and minimal or no symptoms by the other. In those with conflicting severity levels (17%), several descriptive factors were found to predict differences. Being Hispanic or American Indian/Alaskan Native as compared to Caucasian was related to an increased likelihood of differing classification between the BDI‐1A and PHQ‐9. This may relate to the BDI's limited validity in certain racial/ethnic groups as demonstrated by other studies.^12^, ^34^ In addition, a history of CVD and a lower self‐reported cognitive and physical quality of life (MCS/PCS scores) were predictive of conflicting results. In these individuals, administration of only one tool to measure depressive symptoms could paint an incomplete picture. Gaining additional information may add to the clinical picture. Caution should be used in interpreting the results of a single instrument (especially the BDI‐1A) in some ethnic groups.

When considering severity of depression, higher scores on the BDI‐1A predicted differing classification, although these discrepancies seemed to be driven primarily by higher scores on the somatic as opposed to the cognitive subscale. These findings suggest that physical, opposed to psychological, symptoms might have a greater influence on depression screening. Other studies also recognized this difficulty of reporting as well as difficulty in diagnosing depression in the presence of somatic symptoms.^35^, ^36^ This is likely related to several factors. The psychopathology of the somatic component, both painful and nonpainful, of depression is understudied and not completely understood.^37^ For example, lack of pleasure and sleep abnormalities are related to abnormal serotonin and norepinephrine regulation of the hypothalamus and sleep centers, whereas fatigue and loss of energy appear to be affected by malfunctioning neuronal circuits regulated by multiple neurotransmitters.^38^, ^39^ However, the overlapping symptoms of depression and diabetes, particularly somatic symptoms including fatigue, must be considered when these comorbid conditions occur but they are, unfortunately, underrecognized in clinical settings.^1^, ^2^, ^7^, ^8^ Awareness of cognitive and affective symptoms unique to depression (ie, negative thoughts, anxiety) can help clinicians delineate the correct diagnosis of depression.^9^ Furthermore, broad terminology is used in the BDI‐1A to define somatic symptoms such as somatoform, psychosomatic, vegetative, medically unexplained, masked, and somaticized,^36^ making it difficult to differentiate between somatic symptoms related to another psychiatric condition, somatoform disorders, or other medical conditions.^37^ These factors provide rationale for the potential delay of recognition of depression when somatic symptoms are present and may account for discrepancies in reporting. Nevertheless, one meta‐analysis found that two‐thirds of depressed patients reported somatic symptoms,^40^ emphasizing the importance of further investigation for earlier recognition of depression in their presence.

The study findings contrast with a study of low‐income women (18 to 60 years) with one or more chronic health conditions (eg, seasonal allergies, asthma, diabetes, CVD) that failed to find any significant predictors of differences in scores between the BDI‐1A and PHQ‐9.^41^ The conflicting findings are likely related to methodological and study population differences. These analyses compared differences in classification levels that are commonly used in clinical practice, whereas Kneipp et al 41 transformed scale scores into standardized scores (z scores) and compared differences in continuous severity scores. Furthermore, this sample was an older population (mean age 69.8 years) that was more heterogeneous in terms of race/ethnicity, sex, and socioeconomic status.

This study has multiple strengths that include a large racially/ethnically diverse population and the use of well‐validated questionnaires. The focus on a group that has been diagnosed with type 2 diabetes provides specific information for this group; however, these findings may not generalize to individuals without diabetes. A limitation of the current study is that no “gold standard” (eg, a structured clinical interview) was used to determine the presence and severity of depression, with which each of these measures could then be compared. However, the overall goal of this study was to examine differences in reported symptomatology of depression between these two commonly used questionnaires. Additional limitations of the study include low initial rates of depression (ie, 85.9% were categorized as having no or minimal depressive symptoms by the BDI‐1A and 82.1% by the PHQ‐9), low rates of severe depression, and the difference in time periods of reporting between the two instruments (previous one vs two weeks). The findings from this study suggest that future research should compare the differences between the BDI‐1A and PHQ‐9 against a structured clinical interview.

## 5 CONCLUSION

The results suggest that either the PHQ‐9 or BDI‐1A can be used in depression screening for adults with overweight/obesity and type 2 diabetes. The findings suggest the need for further assessment of individuals with type 2 diabetes who have comorbid conditions such as CVD, report greater depression severity (particularly somatic symptoms of depression), have a decreased quality of life, or who are of a certain race or ethnicity (eg, Hispanic or American Indian/Alaskan Native).

## DISCLOSURE(S)

Dr Cheskin reports SAB membership for Plexus Inc and Pressed Juicery Inc. The following organizations have committed to make major contributions to Look AHEAD: FedEx Corporation; Health Management Resources; LifeScan Inc, a Johnson & Johnson Company; OPTIFAST® of Nestle HealthCare Nutrition Inc; Hoffmann‐La Roche Inc; Abbott Nutrition; and Slim‐Fast Brand of Unilever North America.

## FUNDING

This study is supported by the Department of Health and Human Services through the following cooperative agreements from the National Institutes of Health (NIH): DK110341, DK57136, DK57149, DK56990, DK57177, DK57171, DK57151, DK57182, DK57131, DK57002, DK57078, DK57154, DK57178, DK57219, DK57008, DK57135, and DK56992. The following federal agencies have contributed support: National Institute of Diabetes and Digestive and Kidney Diseases; National Heart, Lung, and Blood Institute; National Institute of Nursing Research; National Center on Minority Health and Health Disparities; NIH Office of Research on Women's Health; and the Centers for Disease Control and Prevention. This research was supported in part by the Intramural Research Program of the National Institute of Diabetes and Digestive and Kidney Diseases.

Additional support was received from The Johns Hopkins Medical Institutions Bayview General Clinical Research Center (M01RR02719); the Massachusetts General Hospital Mallinckrodt General Clinical Research Center and the Massachusetts Institute of Technology General Clinical Research Center (M01RR01066); the Harvard Clinical and Translational Science Center (RR025758‐04); the University of Colorado Health Sciences Center General Clinical Research Center (M01RR00051) and Clinical Nutrition Research Unit (P30 DK48520); the University of Tennessee at Memphis General Clinical Research Center (M01RR0021140); the University of Pittsburgh General Clinical Research Center (GCRC) (M01RR000056), the Clinical Translational Research Center (CTRC) funded by the Clinical & Translational Science Award (UL1RR024153) and NIH grant (DK 046204); the VA Puget Sound Health Care System Medical Research Service, Department of Veterans Affairs; and the Frederic C. Bartter General Clinical Research Center (M01RR01346).

## DATA SHARING PLAN

The study protocol, analysis plan, forms, and detailed data description of de‐identified participants from the Look AHEAD trial may be located at the National Institute of Diabetes, Digestive, and Kidney Diseases Central Repository (study start: June 2001; primary completion: September 2012; estimated completion: January 2020): https://repository.niddk.nih.gov/studies/look-ahead/?query=look%20ahead

## Appendix

Look AHEAD Research Group at the end of continuation

Clinical Sites

The Johns Hopkins University: Frederick L. Brancati, MD, MHS^1*^; Jeanne M. Clark, MD, MPH^1^ (Co‐Principal Investigators); Lee Swartz^2^; Jeanne Charleston, RN^3^; Lawrence Cheskin, MD^3^; Richard Rubin, PhD^3^*; Jean Arceci, RN; David Bolen; Danielle Diggins; Mia Johnson; Joyce Lambert; Sarah Longenecker; Kathy Michalski, RD; Dawn Jiggetts; Chanchai Sapun; Maria Sowers; Kathy Tyler.

^*^deceased

Pennington Biomedical Research Center: George A. Bray, MD^1^; Allison Strate, RN^2^; Frank L. Greenway, MD^3^; Donna H. Ryan, MD^3^; Donald Williamson, PhD^3^; Timothy Church, MD^3^; Catherine Champagne, PhD, RD; Valerie Myers, PhD; Jennifer Arceneaux, RN; Kristi Rau; Michelle Begnaud, LDN, RD, CDE; Barbara Cerniauskas, LDN, RD, CDE; Crystal Duncan, LPN; Helen Guay, LDN, LPC, RD; Carolyn Johnson, LPN; Lisa Jones; Kim Landry; Missy Lingle; Jennifer Perault; Cindy Puckett; Marisa Smith; Lauren Cox; Monica Lockett, LPN.

The University of Alabama at Birmingham: Cora E. Lewis, MD, MSPH^1^; Sheikilya Thomas, PhD, MPH^2^; Monika Safford, MD^3^; Stephen Glasser, MD^3^; Vicki DiLillo, PhD^3^; Gareth Dutton, PhD; Charlotte Bragg, MS, RD, LD; Amy Dobelstein; Sara Hannum; Anne Hubbell, MS; Jane King, MLT; DeLavallade Lee; Andre Morgan; L. Christie Oden; Janet Wallace, MS; Cathy Roche, PhD, RN, BSN; Jackie Roche; Janet Turman.

Harvard Center

*Massachusetts General Hospital*: David M. Nathan, MD^1^; Enrico Cagliero, MD^3^; Heather Turgeon, RN, BS, CDE^2^; Barbara Steiner, EdM; Valerie Goldman, MS, RDN^2^; Linda Delahanty, MS, RDN^3^; Ellen Anderson, MS, RDN^3^; Laurie Bissett, MS, RDN; Christine Stevens, RN; Mary Larkin, RN; Kristen Dalton, BS; Roshni Singh, BS.

*Joslin Diabetes Center*: Edward S. Horton, MD^1^; Sharon D. Jackson, MS, RD, CDE^2^; Osama Hamdy, MD, PhD^3^; A. Enrique Caballero, MD^3^; Sarah Bain, BS; Elizabeth McKinney, BSN, RN; Barbara Fargnoli, MS, RD; Jeanne Spellman, BS, RD; Kari Galuski, RN; Ann Goebel‐Fabbri, PhD; Lori Lambert, MS, RD; Sarah Ledbury, MEd, RD; Maureen Malloy, BS; Kerry Ovalle, MS, RCEP, CDE.

*Beth Israel Deaconess Medical Center*: George Blackburn, MD, PhD^1^; Christos Mantzoros, MD, DSc^3^; Ann McNamara, RN.

University of Colorado Anschutz Medical Campus: James O. Hill, PhD^1^; Marsha Miller, MS RD^2^; Holly Wyatt, MD^3^; Brent Van Dorsten, PhD^3^; Judith Regensteiner, PhD^3^; Debbie Bochert; Gina Claxton‐Malloy, RD; Ligia Coelho, BS; Paulette Cohrs, RN, BSN; Susan Green; April Hamilton, BS, CCRC; Jere Hamilton, BA; Eugene Leshchinskiy; Loretta Rome, TRS; Terra Thompson, BA; Kirstie Craul, RD, CDE; Cecilia Wang, MD.

Baylor College of Medicine: John P. Foreyt, PhD^1^; Rebecca S. Reeves, DrPH, RD^2^; Molly Gee, MEd, RD^2^; Henry Pownall, PhD^3^; Ashok Balasubramanyam, MBBS^3^; Chu‐Huang Chen, MD, PhD^3^; Peter Jones, MD^3^; Michele Burrington, RD, RN; Allyson Clark Gardner, MS, RD; Sharon Griggs; Michelle Hamilton; Veronica Holley; Sarah Lee; Sarah Lane Liscum, RN, MPH; Susan Cantu‐Lumbreras; Julieta Palencia, RN; Jennifer Schmidt; Jayne Thomas, RD; Carolyn White; Charlyne Wright, RN; Monica Alvarez, PCT.

The University of Tennessee Health Science Center

*University of Tennessee East*: Karen C. Johnson, MD, MPH; Karen L. Wilson, BSN; Mace Coday, PhD^3^; Beate Griffin, RN, BS; Donna Valenski; Polly Edwards; Brenda Fonda; Kim Ward.

*University of Tennessee Downtown*: Helmut Steinburg, MD^3^; Carolyn Gresham, BSN; Moana Mosby, RN; Debra Clark, LPN; Donna Green, RN; Abbas E. Kitabchi, PhD, MD (retired).

University of Minnesota: Robert W. Jeffery, PhD^1^; Tricia Skarphol, MA^2^; John P. Bantle, MD^3^; J. Bruce Redmon, MD^3^; Richard S. Crow, MD^3^;Scott J. Crow, MD^3^; Manami Bhattacharya, BS; Cindy Bjerk, MS, RD; Kerrin Brelje, MPH, RD; Carolyne Campbell; Mary Ann Forseth, BA; Melanie Jaeb, MPH, RD; Philip Lacher, BBA; Patti Laqua, BS, RD; Birgitta I. Rice, MS, RPh, CHES; Ann D. Tucker, BA; Mary Susan Voeller, BA.

St. Luke's Roosevelt Hospital Center: Xavier Pi‐Sunyer, MD^1^; Jennifer Patricio, MS^2^; Carmen Pal, MD^3^; Lynn Allen, MD; Janet Crane, MA, RD, CDN; Lolline Chong, BS, RD; Diane Hirsch, RNC, MS, CDE; Mary Anne Holowaty, MS, CN; Michelle Horowitz, MS, RD; Les James; Raashi Mamtani, MS.

University of Pennsylvania: Thomas A. Wadden, PhD^1^; Barbara J. Maschak‐Carey, MSN, CDE^2^; Robert I. Berkowitz, MD^3^; Gary Foster, PhD^3^; Henry Glick, PhD^3^; Shiriki Kumanyika, PhD RD, MPH^3^; Yuliis Bell, BA; Raymond Carvajal, PsyD; Helen Chomentowski; Renee Davenport; Lucy Faulconbridge, PhD; Louise Hesson, MSN, CRNP; Sharon Leonard, RD; Monica Mullen, RD, MPH.

University of Pittsburgh: John M. Jakicic, PhD^1^; David E. Kelley, MD^1^; Jacqueline Wesche‐Thobaben, RN, BSN, CDE^2^; Daniel Edmundowicz, MD^3^; Lin Ewing, PhD, RN^3^; Andrea Hergenroeder, PhD, PT, CCS^3^; Mary L. Klem, PhD, MLIS^3^; Mary Korytkowski, MD^3^; Andrea Kriska, PhD^3^; Lewis H. Kuller, MD, DrPH^3^; Amy D. Rickman, PhD, RD, LDN^3^; Rose Salata, MD^3^; Monica E. Yamamoto, DrPH, RD, FADA^3^; Janet Bonk, RN, MPH; Susan Copelli, BS, CTR; Rebecca Danchenko, BS; Tammy DeBruce, BA; Barbara Elnyczky; David O. Garcia, PhD; George A. Grove, MS; Patricia H. Harper, MS, RD, LDN; Susan Harrier, BS; Diane Heidingsfelder, MS, RD, CDE, LDN; Nicole L. Helbling, MS, RN; Diane Ives, MPH; Janet Krulia, RN, BSN, CDE; Juliet Mancino, MS, RD, CDE, LDN; Anne Mathews, PhD, RD, LDN; Lisa Martich, BS, RD, LDN; Meghan McGuire, MS; Tracey Y. Murray, BS; Anna Peluso, MS; Karen Quirin; Jennifer Rush, MPH; Joan R. Ritchea; Linda Semler, MS, RD, LDN; Karen Vujevich, RN‐BC, MSN, CRNP; Kathy Williams, RN, MHA; Donna L. Wolf, PhD.

The Miriam Hospital/Brown Medical School: Rena R. Wing, PhD^1^; Renee Bright, MS^2^; Vincent Pera, MD^3^; Deborah Tate, PhD^3^; Amy Gorin, PhD^3^; Kara Gallagher, PhD^3^; Amy Bach, PhD; Barbara Bancroft, RN, MS; Anna Bertorelli, MBA, RD; Richard Carey, BS; Tatum Charron, BS; Heather Chenot, MS; Kimberley Chula‐Maguire, MS; Pamela Coward, MS, RD; Lisa Cronkite, BS; Julie Currin, MD; Maureen Daly, RN; Caitlin Egan, MS; Erica Ferguson, BS, RD; Linda Foss, MPH; Jennifer Gauvin, BS; Don Kieffer, PhD; Lauren Lessard, BS; Deborah Maier, MS; JP Massaro, BS; Tammy Monk, MS; Rob Nicholson, PhD; Erin Patterson, BS; Suzanne Phelan, PhD; Hollie Raynor, PhD, RD; Douglas Raynor, PhD; Natalie Robinson, MS, RD; Deborah Robles; Jane Tavares, BS.

The University of Texas Health Science Center at San Antonio: Helen P. Hazuda, PhD^1^; Maria G. Montez, RN, MSHP, CDE^2^; Carlos Lorenzo, MD^3^; Charles F. Coleman, MS, RD; Domingo Granado, RN; Kathy Hathaway, MS, RD; Juan Carlos Isaac, RC, BSN; Nora Ramirez, RN, BSN.

VA Puget Sound Health Care System/University of Washington: Steven E. Kahn, MB, ChB^1^; Anne Kure, BS^2^; Edward J. Boyko, MD, MPH^3^; Edward Lipkin, MD, PhD^3^; Dace Trence, MD^3^; Subbulaxmi Trikudanathan, MD, MRCP, MMSc^3^; Elaine Tsai, MD^3^; Brenda Montgomery, RN, MS, CDE; Ivy Morgan‐Taggart; Jolanta Socha, BS; Lonnese Taylor, RN, BS; Alan Wesley, BA.

Southwestern American Indian Center, Phoenix, Arizona and Shiprock, New Mexico: William C. Knowler, MD, DrPH^1^; Paula Bolin, RN, MC^2^; Tina Killean, BS^2^; Maria Cassidy‐Begay, BSND, RND^2^; Katie Toledo, MS, LPC^2^; Cathy Manus, LPN^3^; Jonathan Krakoff, MD^3^; Jeffrey M. Curtis, MD, MPH^3^; Sara Michaels, MD^3^; Paul Bloomquist, MD^3^; Peter H. Bennett, MB, FRCP^3^; Bernadita Fallis, RN, RHIT, CCS; Diane F. Hollowbreast; Ruby Johnson; Maria Meacham, BSN, RN, CDE; Christina Morris, BA; Julie Nelson, RD; Carol Percy, RN, MS; Patricia Poorthunder; Sandra Sangster; Leigh A. Shovestull, RD, CDE; Miranda Smart; Janelia Smiley; Teddy Thomas, BS.

University of Southern California: Anne Peters, MD^1^; Siran Ghazarian, MD^2^; Elizabeth Beale, MD^3^; Kati Konersman, RD, CDE; Brenda Quintero‐Varela; Edgar Ramirez; Gabriela Rios, RD; Gabriela Rodriguez, MA; Valerie Ruelas, MSW, LCSW; Sara Serafin‐Dokhan; Martha Walker, RD.

Coordinating Center

Wake Forest University: Mark A. Espeland, PhD^1^; Judy L. Bahnson, BA, CCRP^3^; Lynne E. Wagenknecht, DrPH^1^; David Reboussin, PhD^3^; W. Jack Rejeski, PhD^3^; Alain G. Bertoni, MD, MPH^3^; Wei Lang, PhD^3^; David Lefkowitz, MD^3^; Patrick S. Reynolds, MD^3^; Denise Houston, PhD^3^; Mike E. Miller, PhD^3^; Laura D. Baker, PhD^3^; Nicholas Pajewski, PhD^3^; Stephen R. Rapp, PhD^3^; Stephen Kritchevsky, PhD^3^; Haiying Chen, PhD, MM^3^; Valerie Wilson, MD^3^; Delia S. West, PhD^3^; Ron Prineas, MD^3^; Tandaw Samdarshi, MD^3^; Amelia Hodges, BS, CCRP^2^; Karen Wall^2^; Carrie C. Williams, MA, CCRP^2^; Andrea Anderson, MS; Jerry M. Barnes, MA; Tara D. Beckner; Delilah R. Cook; Valery S. Effoe, MD, MS; Melanie Franks, BBA; Katie Garcia, MS; Sarah A. Gaussoin, MS; Candace Goode; Michelle Gordon, MS; Lea Harvin, BS; Mary A. Hontz, BA; Don G. Hire, BS; Patricia Hogan, MS; Mark King, BS; Kathy Lane, BS; Rebecca H. Neiberg, MS; Julia T. Rushing, MS; Debbie Steinberg, BS; Jennifer Walker, MS; Michael P. Walkup, MS.

Central Resources Centers

Central Laboratory, Northwest Lipid Metabolism and Diabetes Research Laboratories: Santica M. Marcovina, PhD, ScD^1^; Jessica Hurting^2^; John J. Albers, PhD^3^; Vinod Gaur, PhD.^4^

ECG Reading Center, EPICARE, Wake Forest University School of Medicine: Elsayed Z. Soliman, MD, MSc, MS^1^; Charles Campbell^2^; Zhu‐Ming Zhang, MD^3^; Mary Barr; Susan Hensley; Julie Hu; Lisa Keasler; Yabing Li, MD.

Hall‐Foushee Communications, Inc: Richard Foushee, PhD; Nancy J. Hall, MA.

Federal Sponsors

National Institute of Diabetes and Digestive and Kidney Diseases: Mary Evans, PhD; Van S. Hubbard, MD, PhD; Susan Z. Yanovski, MD.

National Heart, Lung, and Blood Institute: Lawton S. Cooper, MD, MPH; Peter Kaufman, PhD, FABMR; Mario Stylianou, PhD.

Centers for Disease Control and Prevention: Edward W. Gregg, PhD; Ping Zhang, PhD.

Funding and Support

Funded by the National Institutes of Health through cooperative agreements with the National Institute of Diabetes and Digestive and Kidney Diseases: DK57136, DK57149, DK56990, DK57177, DK57171, DK57151, DK57182, DK57131, DK57002, DK57078, DK57154, DK57178, DK57219, DK57008, DK57135, and DK56992. Additional funding was provided by the National Heart, Lung, and Blood Institute; National Institute of Nursing Research; National Center on Minority Health and Health Disparities; NIH Office of Research on Women's Health; and the Centers for Disease Control and Prevention. This research was supported in part by the Intramural Research Program of the National Institute of Diabetes and Digestive and Kidney Diseases. The Indian Health Service (I.H.S.) provided personnel, medical oversight, and use of facilities. The opinions expressed in this paper are those of the authors and do not necessarily reflect the views of the I.H.S. or other funding sources.

Additional support was received from The Johns Hopkins Medical Institutions Bayview General Clinical Research Center (M01RR02719); the Massachusetts General Hospital Mallinckrodt General Clinical Research Center and the Massachusetts Institute of Technology General Clinical Research Center (M01RR01066); the Harvard Clinical and Translational Science Center (RR025758‐04); the University of Colorado Health Sciences Center General Clinical Research Center (M01RR00051) and Clinical Nutrition Research Unit (P30 DK48520); the University of Tennessee at Memphis General Clinical Research Center (M01RR0021140); the University of Pittsburgh General Clinical Research Center (GCRC) (M01RR000056), the Clinical Translational Research Center (CTRC) funded by the Clinical & Translational Science Award (UL1RR024153) and NIH grant (DK 046204); the VA Puget Sound Health Care System Medical Research Service, Department of Veterans Affairs; and the Frederic C. Bartter General Clinical Research Center (M01RR01346).

The following organizations have committed to make major contributions to Look AHEAD: FedEx Corporation; Health Management Resources; LifeScan Inc, a Johnson & Johnson Company; OPTIFAST® of Nestle HealthCare Nutrition Inc; Hoffmann‐La Roche Inc; Abbott Nutrition; and Slim‐Fast Brand of Unilever North America.

Some of the information contained herein was derived from data provided by the Bureau of Vital Statistics, New York City, Department of Health and Mental Hygiene.

______________________________

^1^ Principal Investigator

^2^ Program Coordinator

^3^ Co‐Investigator

All other Look AHEAD staffs are listed alphabetically by site.
